# Empowering Collaboration With Patients: Healthcare Staff's Perspectives on Conditions for Shared Decision‐Making in Swedish Psychiatric Care

**DOI:** 10.1111/jep.70562

**Published:** 2026-08-20

**Authors:** Charlotte Cederborg, Märta Sund Levander, Berit M. Gustafsson

**Affiliations:** ^1^ Department of Psychiatry Rehabilitation and Diagnostics, Psychiatric Clinic Eksjö Sweden; ^2^ Department of Health, Medicine and Caring Sciences Medical Faculty, Linköping University Linköping Sweden; ^3^ Department of Biomedical and Clinical Sciences Medical Faculty, Linköping University Linköping Sweden; ^4^ Department of Psychiatry, Rehabilitation and Diagnostics Child and Adolescent Psychiatry Clinic Jönköping Sweden

**Keywords:** collaboration, personalised care, psychiatric care, relationship, shared decision‐making

## Abstract

**Rationale:**

Shared decision‐making (SDM) is advocated in psychiatric care to facilitate patients' engagement in treatment discussions about their own care. However, applying SDM in practice involves more than simply referring to an advocated method. It is therefore interesting to study healthcare professionals' perceptions and experiences of the conditions necessary for patients to truly participate in SDM.

**Aim:**

To explore healthcare staff's experiences of patient involvement in psychiatric care, paying particular attention to perceived prerequisites, barriers and strategies relevant to shared decision‐making.

**Methods:**

This was an exploratory qualitative study, utilising an inductive approach. Twenty‐eight psychiatric care professionals (21 women and 7 men; nurses (10), nurse's assistants (7), psychologists (2), psychotherapists/therapists (6), psychiatrists (1), junior doctors (1) and peer support (1)) participated in focus group interviews. Data was analysed using conventional content analysis, deriving coding categories directly from the transcripts without the influence of prior theoretical frameworks.

**Results:**

Four main categories emerged: *care relationship*, *patient resources*, *organization* and *strategies in patient meetings*. Staff emphasised *time* as an overarching factor, which affected all the main categories. Trust, openness and mutual respect in the care relationship facilitate collaboration and patient involvement. Staff highlighted personalised care that aligns with patients' resource needs and values, supported by professionalism and transparency in communication. Beyond relational factors, organizational challenges—including time constraints and resource limitations—were barriers to patient involvement in SDM. Strategies such as motivational interviewing, documentation in patient records and care plans, and multidisciplinary teamwork were identified as facilitators.

**Conclusions:**

Overall, the study illustrates that staff work towards a good care relationship to strengthen patients' engagement in SDM in psychiatric care. To truly apply patient involvement and SDM in practice, organizational support is needed, in the form of more time, more available resources and clearer care structures. In addition, organizational aspects, such as integrating patient involvement into system‐level care planning, education and structures for decision‐making processes, could further enhance patient involvement in SDM. Further research is needed to investigate how these perceived states translate into other cultural psychiatric contexts and how they are experienced by patients.

## 1 Introduction

Recent research underscores that involving patients in their own mental health treatment brings benefits, including greater patient understanding of their condition, improved adherence to treatment, enhanced patient satisfaction and potentially better overall outcomes [1, 2, 3]. From an organizational perspective, shared decision‐making (SDM) contributes to lower healthcare costs by reducing the need for inpatient admissions. Additionally, patient satisfaction with the decision‐making process was a predictor of healthcare costs [4].

SDM is a method for increasing patient involvement in their own care regarding decisions, available options, risks/benefits, preferences and shared planning. It is advocated by the National Board of Health and Welfare [5, 6] as a recommended practice to involve patients within mental healthcare in Sweden. SDM conceptualises an intermediary model, bridging the gap between a paternalistic approach, where the physician decides unilaterally based on their expertise, and the informed‐choice model, where the patient decides autonomously based on information provided by the physician. SDM enhances patient autonomy while ensuring active involvement in the decision‐making process, thereby avoiding the extremes of both paternalism and patient isolation in decision‐making [7]. However, as demonstrated by Lee and Gagliardi, SDM should not be used interchangeably with related concepts such as person‐centred care, recovery‐oriented care, or the therapeutic alliance. Although these relational constructs strongly facilitate patient involvement, SDM uniquely refers to the explicit, dyadic process of collaborative deliberation and shared treatment decisions. Consequently, these broader frameworks serve as essential facilitators that underpin—but do not constitute—the specific mechanism of SDM [8].

SDM interventions play a pivotal role in patient involvement in psychiatric care by facilitating discussions between the caregiver and the patient on treatment options, taking into account patient preferences, and considering the advantages and disadvantages of different approaches [2]. Research suggests that SDM can mitigate decisional conflict and enhance self‐esteem, problem‐solving ability and quality of life, and that individuals with severe mental health conditions exhibit positive attitudes towards it, expressing a desire to be actively involved in decision‐making processes [7, 9].

The SDM process in Swedish psychiatric care, as outlined by the National Board of Health and Welfare [6], involves a collaborative effort in which both clinician and service user are conceptualised as experts. Together, they should identify the decision needs, clarify their roles, present options and associated risks, understand each other's values, explore preferences, engage in discussions, achieve mutual consent and plan for follow‐up care. SDM must therefore include trusting collaboration between care providers and care recipients based on an equal relationship. Most patients favoured an active or collaborative role, and a significant proportion preferred to share decision‐making with their clinician, while a smaller percentage wished to make the final decision independently [10]. A minority of patients preferred a passive role in the decision‐making process [10].

Fear plays a significant role in the lives of adult mental health service users, arising from factors like power imbalances, stigma and discrimination. In psychiatric services, these issues have the potential to create a climate that can discourage patients from engaging openly with their care, hindering communication and trust [11]. Feeling seen and understood was important for patients staying in healthcare institutions, and the relationship between the caregiver and the patient formed the foundation for SDM [12]. An integrative review came to the same conclusion: that communication and relationship‐building is foundational for SDM [13]. SDM can thus help to mitigate the obstacles of fear, power imbalance, stigma, etc. Moreover, effective risk management in mental health settings is reliant upon the therapeutic relationship between healthcare professionals and patients [14].

Collaboration between health professionals and patients depends on desire, capability and the time invested by both parties [15]. Poor illness phases pose obstacles to patient involvement due to a lack of insight, verbal ability and/or cooperation [16]. When health professionals perceive patients as unable to participate in SDM, involvement may be reduced to merely providing information about their illness and treatment, assigning the patient a passive role [15]. Demands for compliance to treatment, rules and routines in therapeutic settings, with staff primarily viewing patient involvement as engagement in mandatory activities, can limit patient autonomy [17], as can staff correcting patient demands to fit the existing range of services and organization of care [18]. Mental health professionals perceive collaboration as a challenging yet rewarding process, requiring the adaptation of their knowledge and skills based on patient resources and needs [15]. Hence, they may consider SDM to be intricate and time‐consuming, making it more suitable for long‐term decisions, such as treatment programs, rather than individual, acute decisions [3]. Clinicians often perceive the level of SDM to be higher than patients do, and consultant psychiatrists perceive patients' limited insights into their mental disorder as a significant obstacle to SDM [19, 20].

However, applying SDM in practice involves more than simply referring to an advocated method. A prerequisite for SDM is a mutual exchange of information between healthcare professionals and patients, with deliberation and decision‐making being shared between both parties. A mutual exchange requires a strong alliance, built on trust between patient and professional, mutual information sharing, and respect for the patient's own preferences. It is therefore imperative to explore staff's perspectives in mental healthcare, as this offers insights to complement those derived from the patient's viewpoint [19]. The aim of this study was to explore healthcare staff's experiences of patient involvement in psychiatric care, paying particular attention to the perceived prerequisites, barriers and strategies relevant to shared decision‐making.

## 2 Methods

### 2.1 Design

An exploratory qualitative design, guided by an inductive approach, was utilised to investigate healthcare professionals' perceptions of patient involvement in shared decision‐making within the context of psychiatric care. We used focus group interviews to take advantage of the group dynamics [21, 22, 23]. Focus group interviews generate a rich and nuanced understanding of participants' experiences and perspectives, leveraging group dynamics to encourage discussion and reflection [23]. This approach ensures comprehensive coverage of the majority of potential themes, and facilitates the identification of the most prevalent themes within the dataset [24]. We anticipated that the group setting would encourage participants to recall specific instances or challenges they had encountered in their practice related to SDM [25]. We assumed that professionals working within the same organizational context would feel comfortable and open because the moderator, who was familiar with the clinical environment, could help to establish trust [25]. This approach enabled an efficient and in‐depth exploration of the factors influencing SDM in psychiatric care.

### 2.2 Participants

The inclusion criterion for the convenience sample (*n* = 28) was that participants were permanent employees in clinical inpatient and outpatient psychiatric care. The heterogeneous selection of participants for the focus groups included registered nurses (10), nurse's assistants (7), psychologists (2), psychotherapists/therapists (6), psychiatrists (1), junior doctors (1) and peer support (1). The focus groups consisted of 30 participants, 23 women and 7 men, aged 44.5 ± 9.5 years (range 25–65 years). They had been working in psychiatric care for an average of 11.1 ± 8.5 years (range 0–30 years). Peer support consists of people with experience of mental illness, who are employed to support inpatients.

### 2.3 Setting

The setting is a psychiatric clinic in southern Sweden, which includes five psychiatric outpatient clinics and inpatient care at the county hospital. The clinic is one of three adult psychiatric clinics within the service area of a regional administrative and healthcare entity. The non‐profit clinic serves as a provider of specialised psychiatric care for local residents aged 18 and older. It offers a range of services to address the mental health needs of the local population. Given the region's mix of rural and urban areas, the clinic plays an important role in ensuring access to psychiatric services for individuals in both towns and remote rural communities. The clinic's focus is on delivering comprehensive mental healthcare, tailored to the needs of individuals, across different age groups and conditions. The clinic has a total of 132 permanently employed registered nurses, care assistants, psychologists, psychotherapists, social workers/counsellors, psychiatrists, assistant physicians and peer support. The annual service rate for the hospital's catchment area is 564 inpatient care episodes and 3800 outpatient visits. The researchers MSL and BG have no connection to employers in the clinic, while CC works as a psychologist at one of the included outpatient units.

### 2.4 Data Collection

Between May and September 2024, five focus groups were conducted with staff at the psychiatric clinic, each facilitated by one master's student as moderator (CC) and one trained researcher as observer (BMG). Firstly, the unit managers were informed about the focus groups at a management meeting and were given written information to hand out to staff. These unit managers then informed all permanent employees at formal meetings and via personal email and invited them to participate. After 3 weeks, reminders were sent to the unit managers, who re‐informed their staff. Following this, participants from the various units registered directly with the focus group moderator via mail. The researchers held the focus groups at times convenient for the staff on the ward and at each outpatient psychiatric clinic. All participants filled out a questionnaire providing sociodemographic data about themselves prior to the start of each focus group discussion. After repeating information about the study, the moderator led the interview, which was audio recorded for later verbatim transcription. An interview guide with open‐ended questions constituted the framework for the session [25]. We conducted a pilot group to test the interview guide. No changes were made as a result of the pilot, so the data from the pilot group was included in the final results. The questions were designed to gain a deeper understanding of the staff's experiences, such as ‘Describe what SDM means to you?’, ‘In what ways do you feel that the patients are involved in their care?’, and ‘What do you think about working methods that contribute to increasing patient involvement and SDM?’ The order of the questions was adapted by the moderator to fit with the flow of the group conversation, and probes such as ‘What do you think about that?’, ‘Can you explain more?’, and so on were used to add more depth to the answers. The focus groups lasted between 39 and 61 min. Saturation in the data appeared during the third interview, and was further strengthened with two more interviews.

### 2.5 Data Analysis

A conventional approach to qualitative content analysis was utilised, allowing coding categories to arise directly from the data without being shaped by prior theoretical assumptions [26]. Each focus group constituted a unit of analysis. The initial stage of the analytical process concentrated on the manifest content, which refers to the visible and apparent outcomes [27]. To ensure that the staff's own expressions were emphasised, no predefined codes were used. After three focus groups, no significantly new categories or experiences emerged. We therefore assessed that data saturation had been achieved because the same experiences were emerging among the respondents. The manifest analysis was carried out by the first author (CC), who read the transcripts multiple times to become familiar with the text and grasp the overall meaning. During the second stage, CC and BMG identified and condensed meaning units related to the topic [28]. Given the group discussion format of the data, the researchers also considered meaning units that emerged not only from individual opinions, but also from the group dynamic during the conversation [22]. The condensed meaning units were then coded using labels that emerged directly from the text, and were organised into subcategories. Approximately 5% of the suggested codes were discussed until consensus was reached among the researchers. Subsequently, in the latent analysis, the subcategories were consolidated into mutually exclusive main categories [23, 26, 27] by CC, BMG and MSL. To confirm rigour and transparency in the data analysis, all three authors independently reviewed the material to ensure that coding, subcategories and main categories truly reflected the underlying data. Differences were discussed and compared with the original interview data until agreement was reached. For example, meaning units related to patients' hesitation about expressing needs were initially coded as ‘fear of expressing needs’ and later grouped into subcategories reflecting barriers to involvement. Reflexivity was considered throughout the analysis process by the researchers continuously reflecting upon how their own preconceptions and previous experiences might influence their interpretation of the material. Analytical memos were written at regular intervals and the analysis was discussed repeatedly in the research group to highlight and problematise different interpretations and ensure that the emerging themes were anchored in the participants' statements. In particular, the involvement of one researcher with clinical experience in the study setting may have influenced the data analysis and interpretations. To address this, all three authors engaged in ongoing reflexive discussions, challenging and complementing each other's perspectives to reduce the risk of bias.

The selected quotations were taken directly from the participants' own words contributing to the credibility of the findings. The quotations was translated from Swedish into English. Table 1 illustrates the analysis process of how meaning units, codes and subcategories led to the development of a main category.

**Table 1:** An example of the analysis process: The relationship between meaning unit, codes, subcategories and main category.

Meaning unit	Code	Subcategory	Main category
‘When discussing therapeutic alliances, it always involves these three aspects: goals, methods and relationships. If all three are agreed upon, the alliance works’ ‘We aim to create trust and belief in the person. It's about building a partnership with the patient, working together to achieve the best outcome’ ‘Often, patients know what they need but may not know how to express it’ ‘It's important for patients to be included and valued, ensuring their voices are heard’	Negotiation; Agreement; Shared understanding; Utilise the patient's resources, history and experiences; Partnership; Active and responsible patient	Alliance	
‘I think it's important that they feel trust and dare to open up, to share how they themselves think and feel’; ‘To have trust in each other within the staff group’ ‘If the staff collaborate and communicate between rounds, the patient won't receive conflicting information from different staff members’; ‘The patient feels some form of trust, or that I want to help, that I want to try to make the person feel better’	Earn the patient's trust; Be well‐informed about the patient; Be professional; Collaborate and communicate the same message to the patient	Trust	
‘I strongly believe that the patient needs to understand why we make diagnoses and to discuss it: What does it mean to have that diagnosis? What are the consequences of it? And that they receive information about medication, that there are actually alternatives to choose from’ ‘The minimum standard is, in some way, information—that patients should always know why things are happening and what the caregiver based the decision on’	Informed and knowledgeable about the patient's: difficulties, different interventions, medication, diagnoses	Informed patients	

### 2.6 Ethical Considerations

This study is part of an ongoing project aiming to investigate SDM in psychiatric care interactions at a county hospital psychiatric clinic. The entire study received approval from the Ethics Committee for Human Research at the Medical Faculty of the University of Gothenburg (no. 2022‐00582‐01). Informed consent was obtained in both written and verbal form from all the focus groups participants, in accordance with the Declaration of Helsinki. The participants had the right to withdraw without providing a reason at any time during the study. To ensure confidentiality and anonymity, participants' names were pseudonymized. The researchers had no working or personal relationship with the participants prior to the study. However, the master's student was employed at the same clinic as one of the focus groups, although she had no direct supervisory or managerial relationship with the participants.

## 3 Results

**Figure 1:**
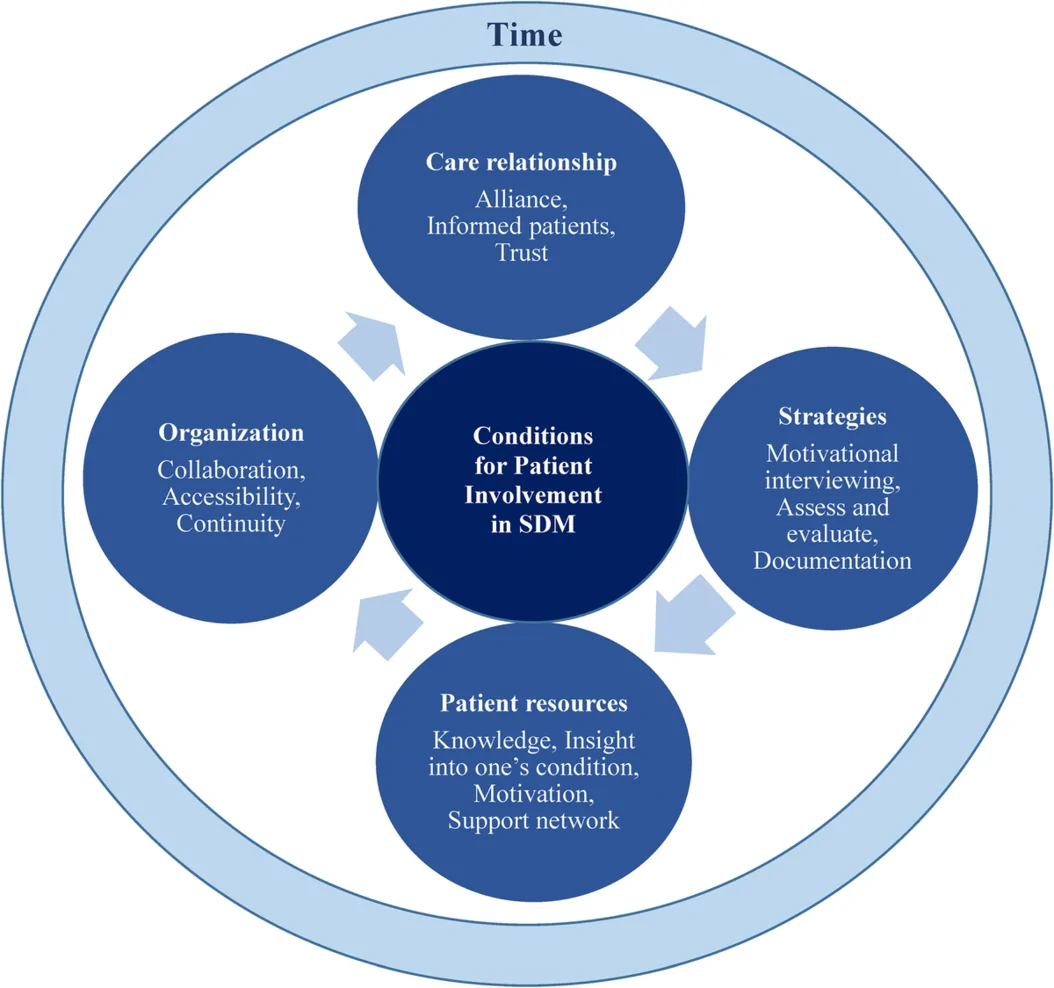
Healthcare staff's experiences of conditions affecting patients' involvement in shared decision‐making (SDM) in psychiatric care.

In the analysis, four main categories emerged as important conditions for patient involvement in SDM: *Care relationship* (with the subcategories Alliance, Informed patients, Trust); *Organization* (with the subcategories Collaboration, Accessibility and Continuity); *Patient resources* (with the subcategories Knowledge, Insight into one's psychiatric condition, Motivation and Support network); and *Strategies* (with the subcategories Motivational interviewing, Assessing and evaluation and Documentation). The staff consistently emphasised Time, which appears to be an overarching factor that affects the four main categories (see Figure 1).

### 3.1 Care Relationship

When developing a care relationship with patients, the respondents emphasised the importance of establishing a patient‐centred interaction, based on equity and collaboration. This process involved recognising and valuing patients' resources and experiences in order to achieve mutually agreed‐upon objectives. Achieving true equity was experienced as difficult, due to the inherent power imbalance between patients and care providers. Respondents viewed the contributions of patients as equally important to those of care providers when selecting methods, interventions and medications, supporting a collaborative dynamic between patients and staff. They believed that the strength of this alliance emerged from the patient's resources, understanding of their condition and willingness to assume responsibility when pursuing shared goals. The respondents highlighted the importance of informing patients about their conditions, because this knowledge empowered them to engage meaningfully in the decision‐making process. They perceived well‐informed patients as being better equipped to ask relevant questions, express their preferences regarding available interventions, and articulate their concerns, thereby enabling a collaborative environment. According to the respondents, this active involvement not only enhanced the quality of decisions but also helped to align treatment plans with the patients' values and goals.

It's important to engage in a pedagogical discussion about the thought processes involved in the diagnostic process, what it means to have that diagnosis, and the associated consequences… and when it comes to treatment, to provide information that there are options to choose from.

(FG1)

Staff emphasised that the ultimate decision rested with the patients and that the requirement to inform patients about available interventions and associated costs was a challenge. The care alliance was considered fundamental to the care relationship, encompassing shared goals, methods and the overall interaction between patients and providers. According to the staff, many individuals reported feeling dependent upon their care providers. The respondents stressed the importance of being aware of this power disparity.

Many patients feel that they're dependent upon their care providers… evening out the power imbalance then makes them feel empowered.

(FG1)

Respondents believed that building an alliance with patients required a coherent care contact along with a trusting and safe relationship. They described the process as one that took time and patience, necessitating stability in the care relationship. The respondents perceived many patients as experiencing significant fear and anxiety during their initial interactions with psychiatric clinics, underscoring the necessity for care providers to earn their trust. They believed that it was common for patients to feel apprehensive about expressing their needs and difficulties right away. Multiple meetings were therefore often required before patients felt secure enough to share their concerns. The respondents explained that demonstrating genuine interest in patients and conveying their value strengthened trust and a sense of safety. Affirmation was described as a tool that helped patients feel seen, heard and understood, thereby creating a safe environment that was conducive to active involvement in their own care.

Our patients are too scared to share their needs and difficulties immediately. We need to meet them several times in order for them to feel safe enough to share. It's a relationship that needs time to grow.

(FG3)

Additionally, respondents stressed that maintaining professionalism and competence—while ensuring openness and transparency about the diagnostic process, the rationale behind treatment recommendations, the effects and side effects of prescribed medications and the costs and benefits of care interventions—was essential for building trust.

…The idea is to choose the right medication together with the patient based on a discussion of pros and cons. Expected effects, common side effects, and so on…

(FG1)

To further establish trust, respondents emphasised the importance of all staff members communicating consistent information regarding interventions, medications, waiting times and other relevant matters to patients.

It's the foundation of care that the patient is on board and actively involved, feeling seen and heard… they should be able to share their experiences and suffering without fear of judgment.

(FG2)

The staff emphasised that a trusting care relationship allowed patients to voice their concerns and dissatisfaction, including issues with their regular care contact. While openness was generally described in terms of information‐sharing and relational safety, concerns that patient involvement may sometimes be more apparent than real also emerged:

Sometimes it might look like there's involvement, but it's not really like that. Patients learn how to survive in the system…

(FG1)

### 3.2 Organization

Team collaboration emerged as a cornerstone condition for SDM. Staff explained that diverse professional perspectives on the patient—considering their problems, resources and needs—were crucial for developing a recommended diagnosis and treatment plan. The care plan was subsequently presented to the patient for discussion, potential adjustments and final agreement. Additionally, the team played a role in revising and re‐evaluating decisions and plans when outcomes were found to be less than optimal. Peer support was regarded as an important asset, serving as a bridge between patients and the healthcare system. Respondents emphasised trust within the care team as vital for encouraging open communication among colleagues and with patients. A collaborative environment in which challenges and difficulties could be openly addressed was considered essential for maintaining open and transparent communication within the team, as well as with patients. These concerns were relayed to the team and discussed without stigma, and potential solutions were developed. These solutions were then shared with the patient, who participated in deciding on the most suitable course of action.

A sense of safety within the psychiatric care team was considered fundamental, with staff noting its direct impact on the quality of care. Confidence in the abilities of team members to provide high‐quality care created a collective assurance that was seen to translate into better outcomes for patients. Collaborating with multiple team members, as opposed to relying on prolonged individual contact, was described as strengthening transparency and trust within the team, which further benefited patient care.

To have many professions sit down with the patient and decide who does what, so that the patient is involved in the planning and decisions… We're all collaborating around the same patient.

(FG4)

When you feel safe in your team and with your teammates, and you know that all your colleagues provide good care, this translates into safety for the patients as well. They read us.

(FG4)

Staff felt that the accessibility of options, such as therapeutic modalities, medical treatments, inpatient care options, and access to staff and therapists, influenced patient involvement in SDM. Sometimes options that the clinic could not offer were omitted, even when these might have been optimal. Respondents felt that this limited patients' ability to make fully informed decisions and compromised the quality of care. Clear communication about these options was seen as crucial to promoting informed and active involvement.

We operate within a system that concerns itself with the following questions: What's available? Do we have anything to offer? Can we meet the patient's needs? We're significantly constrained by the available services.

(FG1)

Accessibility, as discussed within the focus groups, was not limited to clinical services. It also included effective communication between patients and care providers. Additionally, staff believed that various organizational systems, such as telephone‐based queuing systems for managing calls and web‐based health information services, were valuable tools for enhancing patient access to medical advice and information about available healthcare options.

Meeting patients' complex needs was seen as requiring close collaboration with neighbouring care providers, including psychiatric care at various levels, primary care, somatic healthcare and institutions such as social services and local municipalities. Staff across several focus groups shared the perception that psychiatric patients were often neglected by these other providers, with their issues being dismissed as relating solely to mental health. This misinterpretation led to frustration for psychiatric staff and, according to the staff, for the patients as well. Therefore, assisting patients in navigating these systems was described as a crucial part of psychiatric care, underscoring the importance of defining responsibilities between psychiatry and other services while enhancing patient involvement in care.

It's part of our commission to ensure continuity of care… It's part of shared decision‐making to see the whole person, to ensure that there's cooperation between neighboring care providers as well as other social institutions.

(FG4)

### 3.3 Patient Resources

The respondents were of the opinion that patient involvement in SDM was more achievable when patients possessed comprehensive knowledge about and insight into their condition, including its implications, potential consequences and available interventions. Participants believed that information about options boosted patients' confidence and generated a sense of ownership over their care journey. An informed patient was considered more likely to engage in productive discussions, leading to decisions that were more aligned with their needs and preferences, ultimately improving overall health outcomes.

Knowledge and information, I think, are absolutely the most important. Why does it turn out this way for me? If they gain knowledge, they can start to deal with it… They may have had anxiety for many years without anyone explaining what it is…

(FG2)

Respondents emphasised motivation as a factor because it encouraged patients to take an active role in managing their care. A motivated patient was described as being more inclined to engage in meaningful discussions, which led to decisions that aligned with their personal needs and preferences, ultimately enhancing health outcomes. Moreover, motivation was considered crucial for ensuring patient involvement in essential aspects of their treatment, such as attending appointments, engaging in therapeutic interventions and following prescribed medications, all of which were seen as vital for the effectiveness of the overall treatment plan.

The main reason why therapy doesn't work is that the patient isn't motivated. It's not them who wanted this to work. It's someone else, or someone has tried to convince them.

(FG2)

I believe we have a responsibility to motivate people… I think our clinical assessment is based on recognizing a need, and if we don't have the patient on board, I think it's our task to motivate them.

(FG1)

However, participants pointed out that the range of choices available to patients was shaped by the resources and options within the care setting. These decisions included matters such as selecting specific medications or participating in exposure exercises, as well as more everyday choices, such as deciding what to have for dinner on the ward.

The patient needs to be informed about which interventions are available, and also the cost of the intervention for the patient. It's a job that's expected of them, a hard job… In the end, it's their choice.

(FG2)

For me, it's about the patient feeling that they have the opportunity to influence their care, whether that involves taking their walks at one o'clock, doing puzzles in the morning, deciding which medications to test, or whether to receive ECT or TMS, or whatever else it may be. It's important that they're consulted.

(FG5)

Patient involvement often required balancing the individual needs and desires of the patient with the care provider's recommendations. Respondents underscored the necessity of the care provider determining which treatments could be offered based on assessments grounded in knowledge and expertise. Care providers could then assist patients in choosing between options by explaining, in a pedagogical manner, the various treatment alternatives being recommended. Respondents reflected upon how coercive measures directly contradicted the principles of patient involvement in SDM, but were considered necessary when certain psychiatric conditions were present.

Coercive measures create resistance to treatment from the patient, leading to a loss of motivation and the erosion of our alliance with them.

(FG5)

Respondents frequently expressed a desire for people in the patient's support network to be involved in the care process, thereby serving as a resource for both the patient and the healthcare system. Involving these supportive individuals—by having them read the care plan, recognise early warning signs and understand the crisis plan, as well as informing them that they could contact the care provider if they were concerned—was believed to create a calmer environment for the patient, their loved ones and the care provider.

I believe it can take a significant toll on the patient's relationships when they don't share what's happening in their care with their network. I encourage all patients to communicate what we've planned and decided, but there's no guarantee that they will do so.

(FG2)

### 3.4 Strategies

The respondents reported the use of specific strategies during patient meetings to facilitate patient involvement, such as motivational interviewing and open‐ended questions to encourage motivation and engagement. They described motivational interviewing as a way of transferring responsibility for decision‐making to the patient while simultaneously supporting autonomy. Additionally, providing patients with the time and space to engage was another aspect emphasised in the focus groups.

Sometimes I simply take a step back, waiting and listening more. It's important to have patience and provide space for patients to truly engage.

(FG3)

The assessment and evaluation of treatment interventions were considered essential for determining their effectiveness and ensuring that the patient remained engaged and motivated throughout the process. Assessments were common across most therapeutic modalities, providing a platform for the patient to express their thoughts, encouraging them to evaluate various aspects and serving as a tool for the therapist to identify issues that might have been difficult for the patient to articulate. Therapeutic approaches included structured assessments of the alliance between therapist and patient, which staff using these methods found beneficial for enhancing patient involvement.

There's a constant process of alignment: Are we working on what we said we would? Have we reached our desired goals? Should we continue to meet as frequently as we previously discussed?

(FG4)

As part of the strategies, reviewing patient medical records emerged as essential for maintaining an updated understanding of each patient during, and between, interactions. This practice was seen as a tool to enable clinicians to remain informed about the patient's history and issues, ensuring that both outpatient and inpatient care are aligned and that compatible interventions are employed. Focus groups emphasised that staff familiarity with the patient's psychiatric record played a crucial role in enhancing patient engagement. Additionally, the opportunity for patients to access and read their own psychiatric records was described as a means of promoting involvement and keeping patients informed about their care.

… important to read up on the patient's records before meeting them for the first time: Why is the person here? What's happened? How has the process been? When I'm informed about the patient… then they feel heard and listened to!

(FG5)

Yet another strategy was a collaborative care plan designed to empower patients by outlining the responsibilities of both the patient and the healthcare provider. Typically, the care plans were created by the third visit and updated annually. The plan was described by respondents as including goals, strategies, a crisis plan and regular progress reviews. The psychiatric care plan was viewed as a tool for establishing a contract with the patient and to outline a clear plan and objectives for the patient's psychiatric care, considering the patient's problems, resources and goals. Respondents explained that the process of developing the care plan helped patients to identify and articulate their needs. The care plan was seen as particularly beneficial for new patients and specific diagnostic groups who may receive interventions and, ideally, at some point reach a conclusion to their treatment. For patients with chronic diagnoses who seek care and follow‐up only during periods of exacerbated psychiatric symptoms, respondents described difficulty in recognising the value of such a plan.

Respondents also asserted that the psychiatric care plan serves as an overarching framework that broadly specifies the interventions required to address the patient's issues. They contended that it did not contribute significantly to daily treatment practices, and that a concrete implementation plan, or work plan, which could be monitored and evaluated, might have been more useful on a daily basis.

I believe that the care plan is a result of the patient's involvement. If we want to have good care plans that serve their purpose, they should be a result—a symptom—of our work in which the patient is engaged.

(FG1)

According to the respondents, the care plan was useful for facilitating communication between outpatient and inpatient psychiatric care, and it was also beneficial for the patient to review their plan. However, care plans could be monitored and evaluated by management as an issue, which resulted in the risk of psychiatric care plans becoming mere token metrics. This, in turn, might lead staff to create care plans, not for the benefit of the patients themselves, but due to pressure from management and criticism received for insufficient completion of these plans. When this occurred, the fundamental concept of the care plan being a product of the patient's involvement in their own care was undermined, leading to irritation and resistance among staff.

A good care plan outlines what needs to be done, but often we haven't yet captured the essence of the issues when it's written. There needs to be a written document to create the appearance of patient involvement in care in order to make the statistics look favorable.

(FG3)

The study also revealed the importance of staff acting in a supportive capacity to ensure that patients could engage fully in decisions, plans and interventions. Simplifying and clarifying information to compensate for certain patients' disabilities was highlighted as essential. This included helping patients to retain and recall information from meetings, as well as assisting them in formulating questions and expressing preferences within the care relationship.

I need to formulate and deliver the care I offer in a way that's tailored to the individual sitting in front of me.

(FG3)

### 3.5 Time

Respondents generally viewed a lack of time as an obstacle to promoting patient involvement and SDM. It was described as essential to have enough time for caregivers to fully understand patient needs, build trust and develop open communication. Respondents explained that dedicating sufficient time enabled the development of a strong therapeutic alliance and supported an ongoing exchange of information.

It requires patience. It must take the time needed until the patient begins to feel trust and security with the therapist.

(FG2)

Time was also considered necessary for effective team collaboration, enabling the integration of diverse professional perspectives in diagnosing and planning treatment. Detailed discussions with the patient, adjustments to treatment plans and final agreements all required adequate time. Continuous revision and re‐evaluation of plans depended on there being sufficient time for collaboration within the team and with the patient. Being successful in meeting patients' complex needs, according to respondents, depended on having the time to develop effective collaboration across various healthcare sectors, including psychiatric care, primary care, somatic healthcare and social services. Coordination across these sectors was time‐intensive but necessary to ensure comprehensive care. In terms of accessibility, respondents regarded time as crucial for establishing reliable communication channels. Systems such as regular care contact, phone queues and web‐based services took time to set up and maintain. These structures were vital for facilitating quick and effective access to medical advice and information.

To be involved, one [the patient together with their caregiver] needs time. This is a dilemma in healthcare when the influx of patients is large. There are not unlimited resources for everything that needs to be done.

(FG4)

Patient resources, such as knowledge and insight into their own condition, were also linked to the time available. Respondents explained that it took time to help patients identify challenges in ways they could understand. Both caregivers and patients needed time to fully comprehend and address these challenges. While caregivers were believed to develop attentiveness, receptiveness and responsiveness over time, patients were believed to require time to reflect upon and articulate their concerns.

It's essential to understand, alongside the patient, what the core issues are… and that requires more time.

(FG1)

The respondents also highlighted the importance of time in strategies used during patient meetings. Sufficient time allowed caregivers to give patients space, to wait and to listen. This was particularly important for patients who were unaccustomed to articulating their needs or being involved in SDM processes.

Many patients don't know what they feel or need, as they've never been able to meet their own needs. They've been used to not being involved since childhood… and then trying to be attentive and listen to that. It can take a long time.

(FG1)

## 4 Discussion

The results presented here illuminate healthcare professionals' perceptions and what they perceive as the opportunities and barriers to facilitating patient involvement in SDM in a Swedish psychiatric context. The care relationship, organization, patient resources, strategies and time emerged as important factors for patient involvement. Although these relational qualities do not in themselves constitute SDM, participants consistently described them as necessary preconditions for engaging patients in SDM. The results also indicate an awareness of the limitations of current practice and suggest that, despite being valued as necessary for patient involvement, trust and openness may be limited by systematic structures, routines and severe psychiatric conditions.

### 4.1 Care Relationship: The Foundation of Patient Involvement

Our study highlights that staff found a good care relationship to be essential for successfully involving patients in psychiatric care. They emphasised that trust, openness and a sense of safety in the care relationship can strengthen collaboration and mutual respect between staff and patients [7, 29]. In line with other research [12, 30, 31], the present results reveal that trust can be built through affirmation, respect and genuine interest, which helps the patient feel seen, heard and valued.

The present findings highlighted relational engagement—alongside personalised care that considers patients' individual needs, preferences and values—as foundational to building a good care relationship. This aligns with previous findings that patients require professionalism and transparency from caregivers in order to establish a trusting relationship [12, 30, 32]. However, in the present results, openness and trust were consistently emphasised as ideals, while self‐reflection upon how these values were enacted in practice was limited. Barriers were attributed to systemic factors rather than to professional approaches. Some comments suggested an awareness that patient involvement may sometimes be performative, shaped by the need to conform to institutional expectations. The respondents generally expressed a desire for patients to be active participants in their care and emphasised the importance of patient‐centred interactions based on equity and collaboration. This approach involves recognising and valuing patients' own resources and experiences in the work towards mutually agreed‐upon objectives.

In contrast to the present results, Kortteisto et al. [33] found that professionals with more experience, or those in outpatient care, held more positive views on patient involvement. One study [20] found that medical professionals, including psychiatrists, often face structural barriers, such as role definitions and time constraints, while nurses generally rate patient involvement in clinical decisions more favourably than physicians [20]. Interestingly, a distinction emerged in the language used by staff: nurses specifically referred to the care relationship in terms of patient‐centred interactions, whereas other professionals used words such as alliance, highlighting aspects such as trust and respect. Structural barriers may explain some variations in how staff approach and conceptualise their relationships with patients, while the emphasis on relationships when training nurses may explain their use of language emphasising this aspect [34].

Wärdig et al. [35] describe a service user's view of involvement as being listened to, engaging in reciprocal dialogue, and learning about one's health and symptom management. These elements of involvement closely align with our findings, where staff emphasised that open communication and attentiveness are key to developing active patient involvement, an aspect viewed as essential for a strong care relationship. This contrasts with Molina‐Mula and Gallo‐Estrada's [29] findings, which suggest that patients needed to adopt a passive and submissive role in order to establish a good relationship with their caregivers.

The present study also found that staff believed that patients often experience fear and anxiety during initial interactions with psychiatric clinics, feeling dependent upon their care providers. Previous studies have shown that fear and anxiety, combined with perceived dependence upon caregivers, may pose a risk of power imbalances and paternalism within psychiatric care [11, 29, 30]. Respondents therefore emphasised continuity of care, which aligns with findings that fear of losing control, stigma and power imbalances can prevent patients from building the trust needed for genuine collaboration [11, 36]. According to Ocloo et al. [37], both patients and healthcare providers need to be empowered if they are to participate actively in care processes. This includes equipping staff with the tools to support patient involvement and ensuring that patients feel confident expressing their needs [37].

### 4.2 Organizational Factors: Accessibility and Collaboration

Our findings underline the pivotal role of organizational structures in supporting patient involvement. Respondents emphasised that the accessibility of diverse interventions, such as therapeutic methods, medical treatments and inpatient care, is critical for ensuring and supporting opportunities for patients to make their own decisions. In accordance with Agledahl et al. [38], this study argues that patient choice is often constrained by what is medically and organizationally feasible, and that many decisions are never presented to patients at all. This reflects the ambivalence that professionals might experience in care planning as they strive to balance patient demands with organizational constraints and the services provided [18]. Thus, the ways in which patient involvement is implemented may be influenced less by national guidelines [5, 6] and more by institutional boundaries and professional judgement. Staff in the present study believed that patients are more likely to engage in SDM when they have reliable access to mental health services, peer support and institutional resources. However, organizational constraints, such as time pressures and resource limitations, frequently hinder these efforts [39]. Hence, institutional rules that prioritise efficiency over individual needs might hinder patient autonomy and relational engagement, further emphasising the need for strong organizational structures to support collaboration and resource availability [17, 37].

Respondents emphasised that multidisciplinary collaboration is crucial for ensuring accurate diagnoses, consistent and coordinated care, and the development of effective treatment plans. These findings align with those of others, who showed that coordinated care frameworks support patient involvement through collaboration among service providers, and underscore the importance of teamwork, leadership and resource allocation in supporting patient involvement in SDM [39, 40]. Kortteisto et al. [33] suggest a broader conceptualization of user involvement and argue that patients can contribute not only to decisions about their own care but also to clinical guideline development, staff and student education and research. Expanding this understanding and application to include planning, organizational decisions and education could provide opportunities to strengthen patient involvement and align practice with the broader principles of SDM [41, 42].

### 4.3 Patient Resources

Our findings highlight the importance of support networks, insight and motivation as patient resources. Staff consistently emphasised that strong connections with family, friends, or peer support boost patients' confidence in making treatment decisions, provide valuable perspectives, and support them during times of deterioration. This aligns with research emphasising the role of open communication and collaboration, particularly in family‐oriented cultures where collective decision‐making often involves the patient's broader network [40]. However, systemic barriers such as stigma and gaps in post‐hospital care can weaken these networks and reduce patient engagement [11, 30].

Participants in the present study perceived that patients with greater insight and motivation were better able to participate in discussions about treatment options. They argued that a patient's lack of insight into psychiatric conditions may be an obstacle to their involvement. Our findings emphasise that gaining insight requires continuous support and tailored educational efforts to meet patients' evolving and fluctuating needs. Rimondini et al. [14] stress that education and trust‐building are essential to enhance patient insight. Van Der Ploeg‐Dorhout et al. [12] found that patients who feel a sense of ownership over their care and view SDM as both a right and a responsibility are more likely to engage actively in their own care. Molina‐Mula and Gallo‐Estrada [29] argue that patient involvement should be viewed as an ongoing, collaborative process that respects each patient's unique journey.

While SDM is increasingly recognised as a cornerstone of person‐centred psychiatric care, it is complex in the context of severe mental illness [42, 43], which reduces motivation and limits patients' ability to participate [14]. Decision‐making capacity may also fluctuate over time, and situations involving acute psychosis, suicidal tendencies, or compulsory treatment may require temporary restrictions on patient autonomy to ensure safety [44]. Several participants in the present study also described dependence upon healthcare professionals and asymmetrical power relationships as unavoidable features of psychiatric care. These findings suggest that SDM should not be understood as an idealised model in which power is always equally shared, but rather as a flexible and relational process that seeks to maximise patient involvement within the constraints imposed by clinical, ethical and legal realities. It is important to recognise these tensions in order to develop realistic expectations regarding applied SDM in psychiatric practice [45].

### 4.4 Strategies Facilitating Patient Involvement

Wampold and Flückiger [32], along with Cartwright et al. [40], emphasise the importance of building therapeutic alliances. Our study identifies several strategies for doing so, including motivational interviewing, creating space and utilising patient records and care plans. Motivational interviewing, as highlighted by Bischof et al. [46], enhances patient engagement and treatment adherence by developing mutual understanding through active listening and reflection. In psychiatric settings, where patients often experience ambivalence or resistance [11], motivational interviewing can support behavioural change while mitigating communication barriers such as differing communication styles and complex medical language [18, 47].

Motivation for patients to express concerns, preferences, and values also emerged in our study as a critical strategy for promoting autonomy and collaboration. This aligns with Cartwright et al. [40] and Hamann and Heres [7], who emphasise the importance of shared dialogue in supporting collaborative decision‐making. However, Sweeney et al. [11] highlight that fear of judgement or punitive consequences can inhibit patients from engaging openly, underscoring the need for a safe and supportive environment. Systemic challenges, such as organizational barriers, resource limitations and rigid treatment protocols, further restrict opportunities for meaningful collaboration [20, 41].

Reviewing patient records ensures alignment between outpatient and inpatient care while improving trust and engagement, as emphasised in our study. This resonates with Joosten et al. [3] and Eldh [48], who stress that care planning must reflect patient preferences to ensure meaningful involvement. In line with Rimondini et al. [14], respondents also considered patient access to their own medical records to be crucial for patient involvement. However, the respondents in the present study observed that care plans often become token metrics driven by managerial oversight. Also, although useful for new patients, care plans were considered less relevant for those with chronic conditions, reflecting the need for dynamic adaptation. To maximise their potential, care plans should prioritise flexibility, transparency and collaboration while avoiding administrative tokenism [14].

Regular assessment and evaluation of patients' needs emerged as important in our study, ensuring that treatment decisions remain relevant to evolving conditions and preferences. This iterative approach could support engagement, trust, and the role of continuous evaluation in improving satisfaction and adherence [3]. Structured assessments also help to uncover unspoken concerns, particularly in the case of patients who are hesitant to articulate their needs [7, 40, 41]. However, assessments must remain patient‐centred to avoid becoming overly standardised and impersonal [20]. Eldh [48] further emphasises that assessments should reflect patient preferences, serving as tools for engagement rather than compliance.

### 4.5 Time in Shared Decision‐Making

The respondents described time constraints as a major challenge, especially during patient meetings to support reflection, the articulation of needs and active involvement. This is consistent with the findings of Verwijmeren and Grootens [49] and Holzhüter et al. [50], who emphasise the negative impact of limited time on patient engagement and professional training. Building therapeutic alliances, especially with patients who are struggling to understand their conditions, requires sustained time and effort [29, 51]. Time also facilitates iterative care plan adjustments and relational engagement [7, 30].

The respondents in our study described challenges in terms of systemic and organizational resource limitations, in alignment with the findings of Cartwright et al. [40] and Verwijmeren and Grootens [49]. In the present study, respondents emphasised team‐based SDM processes, such as integrating diverse perspectives and ensuring continuity of care, sustained time investment to facilitate team discussions and professional perspectives as a potential way to improve care plans.

However, other findings suggest that SDM can be effective without extending consultation times if adequate support systems are in place [5]. While SDM is often perceived as time‐intensive, Aoki [52] and Légaré and Thompson‐Leduc [53] challenge this assumption, suggesting that systemic inefficiencies rather than SDM itself amplify these perceptions, particularly in outpatient settings with high caseloads [4].

## 5 Limitations

A strength of this study is that it reflects the obstacles and opportunities experienced by staff in clinical practice seeking to fulfil the intentions for patient involvement in psychiatric care. The results can form a basis for improvements in clinical practice. The latent analysis—i.e. the interpretation of results—was conducted by all three authors, reducing the risk of investigator bias, with the researchers supplementing and contesting each other's readings, corresponding to reflexivity [54]. Trustworthiness was enhanced throughout the study by applying strategies to support credibility, dependability, confirmability and transferability. The data analysis was conducted iteratively, with movement back and forth between the transcripts, codes, and emerging themes to ensure that the findings remained grounded in the participants' accounts. The analysis was discussed regularly within the research team, allowing interpretations to be challenged, refined, and supported by the data. Throughout the analytical process, the researchers reflected upon how their pre‐understanding and professional backgrounds might influence the interpretation of the data. Analytical decisions, including the development and refinement of codes and themes, were documented to enhance transparency and dependability. To facilitate transferability, the study context, participants and analytical process are described in sufficient detail to allow readers to assess the applicability of the findings to other settings [55].

Despite efforts to organise focus groups at convenient times, only two physicians participated, which might affect credibility due to selection bias. The sample included few men, reflecting the predominance of women working in psychiatric care. However, this may have limited the diversity of perspectives represented in the study, affecting credibility. Sweden's national guidelines for psychiatric care [6] are based on international recommendations from the National Institute for Health and Care Excellence (NICE). This strengthens the transferability of the results to other clinics with similar psychiatric care systems. However, the organization of psychiatric care, staffing structures, legal frameworks, and patient autonomy vary considerably across countries and healthcare systems, and we therefore argue that caution is warranted regarding the applicability of these findings beyond the local institutional context. The study was not designed to compare perspectives between professional groups. However, we acknowledge that professional roles, responsibilities and decision‐making authority may influence how staff perceive the conditions for patient involvement in SDM.

Another potential limitation is that the participants were all colleagues from the same clinic, which may have influenced group dynamics. While pre‐existing relationships can promote openness and shared understanding, they may also lead participants to present themselves in a more favourable light or to align with perceived normative views, for instance emphasising patient‐centred ideals. Despite these possible effects, the study provides valuable insights into staff experiences and their implications for shared decision‐making in psychiatric care.

## 6 Conclusions

Overall, the study illustrates the ways in which staff work towards a good care relationship to strengthen patients' engagement in SDM in psychiatric care. To truly apply patient involvement and SDM in practice, organizational support is needed, in the form of more time, increased resources, and clearer care structures. In addition, organizational aspects, such as integrating patient involvement into system‐level care planning, education and structures for decision‐making processes, could further enhance patient involvement in SDM. Further research is needed to investigate how these perceived states translate into other cultural psychiatric contexts and how they are experienced by patients.

## Author Contributions

Berit M. Gustafsson, Märta Sund Levander and Charlotte Cederborg designed the study. Charlotte Cederborg and Berit M. Gustafsson conducted the focus groups, and Charlotte Cederborg performed the initial data analysis. All authors contributed to data interpretation and manuscript preparation, and approved the final version.

## Funding

The authors have nothing to report.

## Conflicts of Interest

The authors declare no conflicts of interest.

## Data Availability

The data supporting the findings of this study is available from the corresponding author upon reasonable request.
